# Association Study of the 5′UTR Intron of the *FAD2-2* Gene With Oleic and Linoleic Acid Content in *Olea europaea* L.

**DOI:** 10.3389/fpls.2020.00066

**Published:** 2020-02-13

**Authors:** Amelia Salimonti, Fabrizio Carbone, Elvira Romano, Massimiliano Pellegrino, Cinzia Benincasa, Sabrina Micali, Alessandro Tondelli, Francesca L. Conforti, Enzo Perri, Annamaria Ienco, Samanta Zelasco

**Affiliations:** ^1^ Research Centre for Olive, Citrus and Tree Fruit, CREA, Rende, Italy; ^2^ Research Centre for Olive, Citrus and Tree Fruit, CREA, Roma, Italy; ^3^ Research Centre for Genomics and Bioinformatics, CREA, Fiorenzuola D’Arda, Italy; ^4^ Department of Pharmacy, Health and Nutritional Sciences, University of Calabria, Rende, Italy; ^5^ Research Centre for Forestry and Wood, CREA, Arezzo, Italy

**Keywords:** *FAD2-2* gene, SNP, association study, 5′UTR intron, *Olea europaea*

## Abstract

Cultivated olive (*Olea europaea* L. subsp. *europaea* var. *europaea*) is the most ancient and spread tree crop in the Mediterranean basin. An important quality trait for the extra virgin olive oil is the fatty acid composition. In particular, a high content of oleic acid and low of linoleic, linolenic, and palmitic acid is considered very relevant in the health properties of the olive oil. The oleate desaturase enzyme encoding-gene (*FAD2-2*) is the main responsible for the linoleic acid content in the olive fruit mesocarp and, therefore, in the olive oil revealing to be the most important candidate gene for the linoleic acid biosynthesis. In this study, an *in silico* and structural analysis of the 5′UTR intron of the *FAD2-2* gene was conducted with the aim to explore the natural sequence variability and its role in the gene expression regulation. In order to identify functional allele variants, the 5′UTR intron was isolated and partially sequenced in 97 olive cultivars. The sequence analysis allowed to find a 117-bp insertion including two long duplications never found before in *FAD2-2* genes in olive and the existence of many intron-mediated enhancement (IME) elements. The sequence polymorphism analysis led to detect 39 SNPs. The candidate gene association study conducted for oleic and linoleic acids content revealed seven SNPs and one indel significantly associated able to explain a phenotypic variation ranging from 7% to 16% among the years. Our study highlighted new structural variants within the *FAD2-2* gene in olive, putatively involved in the regulation mechanisms of gene expression associated with the variation of the content of oleic and linoleic acid.

## Introduction

Cultivated olive (*Olea europaea* L. subsp. *europaea* var. *europaea*) is the most ancient and spread tree crop in the Mediterranean basin. Despite the economic, cultural, and ecological importance of olive groves in the Mediterranean area, now extending to other regions, olive has been a poorly characterized species at genetic and genomic level compared to other fruit tree crops. The species is characterized by a very big genome size (1C = 1,400–1,500 Mbp) (Loureiro et al., 2007; Unver et al., 2017), a cross-pollinating reproductive biology leading to a high heterozygosity (Dìez et al., 2011; Besnard et al., 2014; Kaya et al., 2016) and a long generation time. All these aspects, together with the scarce knowledge about the inheritance of most genes controlling agronomical performance and quality traits, have severely restricted breeding strategies to clonal or varietal selection (Rugini et al., 2011). Understanding the basis of quantitative traits may help plant breeders to improve crop yields, resistance to abiotic and biotic stress conditions, end-use quality, and other important characteristics that are controlled by multiple genes exhibiting a quantitative distribution of phenotypes (Kaya et al., 2016). Loci controlling quantitative traits can be identified either by QTL mapping in a biparental segregating population or by association mapping (Belò et al., 2008) in natural populations (Flint-Garcia et al., 2003). Until now, in olive several genetic maps have been built (De la Rosa et al., 2003; Wu et al., 2004; El Aabidine et al., 2010; Dominguez-Garcia et al., 2011; Atienza et al., 2014; Ipek et al., 2016; Marchese et al., 2016) aimed to detect QTL-associated markers for traits, such as fruiting (Ben Sadok et al., 2013; Atienza et al., 2014), ﬂowering (Ben Sadok et al., 2013), trunk diameter (Atienza et al., 2014), and fatty acid composition (Hernández et al., 2017) using different molecular markers and approaches. However, biparental QTL mapping has many limitations in tree species due to their long generation times and juvenile period, high levels of heterozygosity, time-consuming trait evaluation, slow physiological maturation, and high levels of genetic variation between parents (Tian et al., 2014; Kaya et al., 2016).

In recent years, association mapping (AM) methods have been developed to detect the correlations between genotypes and phenotypes on the basis of linkage disequilibrium (LD) (Rafalski, 2010). AM has been a part of research on complex traits in various fruit trees, including peach (Aranzana et al., 2010; Cao et al., 2012), apricot (Olukolu, 2010), sweet cherry (Ganopoulos et al., 2011; Khadivi-Khub, 2014), almond (Kadkhodaei et al., 2011; Font i Forcada et al., 2015), grapevine (Barnaud et al., 2010) and apple (Kumar et al., 2013).

Olive core collection construction has been reported (Belaj et al., 2012; El Bakkali et al., 2013) and very recently new wide SNP polymorphisms in the whole genome and candidate genes were discovered (D’Agostino et al., 2018; Belaj et al., 2018; Cultrera et al., 2019). However, genome data are still incomplete and referred to a few genotypes (Cultrera et al., 2019) while several transcriptomic experiments have been already conducted leading to new candidate genes (García-López et al., 2014; Leyva-Pérez et al., 2014; Carmona et al., 2015; Guerra et al., 2015; Koudounas et al., 2015; Gómez-Lama Cabanás et al., 2015; González-Plaza et al., 2016; Iaria et al., 2015; Alagna et al., 2016; Grasso et al., 2017; Leyva-Pérez et al., 2018). Attempts of genome wide association studies were conducted using molecular markers such as SSR, AFLP, RFLP, and SNP (Ipek et al., 2015; Kaya et al., 2016; Ben-Ayed et al., 2017). However, until high level of genome sequencing information will be available and a whole oriented genome obtained, a candidate gene association mapping seems the most promising approach.

An important quality trait for the extra virgin olive oil is the fatty acid composition. In particular, a high content of oleic acid and low on linoleic, linolenic, and palmitic acid is considered very relevant in the health properties of the olive oil (Quintero-Florez et al., 2015). Recently, it has been shown that dietary supplementation with oleic acid reduces intestinal inflammation and tumor development in mice (Ducheix et al., 2018). In olive, oleic acid content ranges from 57% to 78%, while linoleic acid varies between 7% and 19% (Salas et al., 2000). A significant negative correlation exists between oleic and linoleic acid content (Sabetta et al., 2013; Hernández et al., 2017) since linoleic acid is directly formed by desaturation of oleic acid, which is catalyzed by the oleate desaturase activity (Shanklin and Cahoon, 1998). To date, oleate desaturase encoding gene (*FAD2*) has been isolated and characterized from many plant species, such as rapeseed (Yang et al., 2012), soybean (Heppard et al., 1996; Li et al., 2007), sunflower (Hongtrakul et al., 1998; Martínez-Rivas et al., 2001), peanut (Jung et al., 2000; Chi et al., 2011), flax (Krasowska et al., 2007), safflower (Guan et al., 2012a; Guan et al., 2012b; Cao et al., 2013), sesame (Jin et al., 2001), and cotton (Liu et al., 1999; Zhang et al., 2009). Arabidopsis has only a single *FAD2* gene (Okuley et al., 1994), while most of other plant species possess small or large gene families in which each member is specifically or constitutively expressed in different organs. For example, in grape, *FAD2* is encoded by a small *FAD2* gene family with two members (Lee et al., 2012), while in safflower the *FAD2* gene family is unusually large with 11 functionally diverse members (Cao et al., 2013).

In olive, two genes encoding microsomal oleate desaturases (*OepFAD2-1* and *OepFAD2-2*) have been described and well characterized (Hernández et al., 2005; Hernández et al., 2009; Hernández et al., 2011), whereas only one gene corresponding to the chloroplast oleate desaturase (*OeFAD6*) has been reported (Banilas et al., 2005; Hernández et al., 2011). The *FAD2-2* gene has been considered the main responsible for the linoleic acid content in the olive fruit mesocarp until now (Hernández et al., 2009) but recently in wild olive (*Olea europaea* L. subsp. *europaea* var. *sylvestris*), usually named oleaster, five *FAD2* genes were found (Unver et al., 2017). These authors named *FAD2-3* to the previously characterized *FAD2-2* gene (Hernández et al., 2005; Hernández et al., 2009).

The *FAD2* gene is the most important candidate gene for the linoleic acid biosynthesis in other species as well (Okuley et al., 1994; Belò et al., 2008; Singh et al., 2009; Guan et al., 2012a; Guan et al., 2012b; Font i Forcada et al., 2012);. Several studies focused on this key gene in order to modify the enzyme activity for enhancing the oleic acid content through natural or induced mutations in different species (Tanhuanpää et al., 1998; Hu et al., 2006; Mroczka et al., 2010; Yang et al., 2012; Wells et al., 2014).

However, in the cultivated olive studies aiming to evaluate natural allele variation in modulating the fatty acid composition are still scarce (Ipek et al., 2015; Ben-Ayed et al., 2017; Cultrera et al., 2019). Hernández et al. (2017) found the co-localized QTLs for oleic and linoleic acids, as well as for monounsaturated and polyunsaturated fatty acids, and for the oleic/linoleic ratio in linkage group 20 of Arbequina cultivar. However, the authors did not individuate a single segregating locus controlling the biosynthesis of oleic and linoleic acids. Fine-mapping of this QTL region and the analysis of sequence data are needed in order to highlight the genetic-molecular mechanism underlying the intra-specific natural variation of fatty acid composition in olive oil.

Most of *FAD2* genes isolated in several plant species carry out in their 5′-UTR a large intron, which plays a role in the enhancement of *FAD2* gene expression (Kim et al., 2006; Mroczka et al., 2010; Xiao et al., 2014; Zeng et al., 2017). In sesame, *cis*-elements having a role for the intron-mediated enhancement of *FAD2* gene expression and the promoter-like activity of the intron sequence were identified. The sesame and Arabidopsis *FAD2* introns conferred up to 100-fold enhancement of GUS expression in transgenic tissues of *Arabidopsis* as compared with intron-less controls (Kim et al., 2006).

To clarify the molecular mechanism underlying the natural variation of oleic and linoleic acid content in olive tree species, the *FAD2* 5′UTR intron was analyzed in this work through a bioinformatic, structural, and association study conducted in 97 olive varieties.

## Materials and Methods

### Plant Materials

The research was carried out at the CREA Research Centre for Olive, Citrus, and Tree Fruit official olive tree collection located in Mirto Crosia, Cosenza, Italy on the Ionian coast (39° 37′ 00′′ North latitude, 16° 45′ 53′′ East longitude) at 6 m a.s.l. Olive trees were planted since 1997 with four to five replicates for each variety spaced with a regular planting pattern of 4 × 6 m. The collection maintains more than 500 olive cultivars and accessions collecting from other official collections and commercial nurseries. The olive trees are grown using a vase training system, pruned with a turn of 3 years and usually irrigated during the summer with 1200 mc/ha on average using a localized drip irrigation system. Soil management is mainly characterized by permanent grass. All the cultivars here studied come from Italy with different regional origin (**Table S1**).

### Phenotyping

A set of 97 olive varieties was chosen in order to cover the largest range of the phenotypic variability of fatty acid composition into the olive germplasm available into the collection (**Table S1**). Samplings of 10 to 15 kg drupes at the initial stage of veraison, were carried out from 1 or 2 replicate for each cultivar from 2003 to 2007 and olive oil was extracted within 6/12 hours from harvest using a hammer mill “Oliomio 50” (Toscana Enologica Mori). Olive oil samples were packed in 250-ml dark bottles and stored in a fresh place until analysis. The determination of fatty acid composition was evaluated according to the European Commission Regulation. The fatty acid methyl esters (FAMEs) were prepared following the method described by Christie et al. (1998). FAMEs were obtained by treating 0.15 g of oil with 100 μl of a methanolic solution of 2N potassium hydroxide and n-hexane to make up a final volume of 1.5 ml. The resulting solution was shacked vigorously for 5 min at room temperature. Afterwards, an aliquot of the supernatant (0.2 μl) was dissolved in n-hexane to make up a final volume of 2 ml from which 20 µl were injected into a gas chromatographer (GC). The analyses were conducted by means of an Agilent GC (6890N) equipped with a capillary column SP-2340 (60 m × 0.25 mm i.d., 0.2 μm f.t., Supelco) and a flame ionization detector (FID). Nitrogen was used as carrier gas. The temperature of the column, injector, and detector were set at 180°C, 230°C, and 260°C, respectively. The separation of the analytes was carried out by programming the temperature as follows: 110°C held for 5 min, increase of 3°C/min to 150°C and held for 16 min, increase of 4°C/min to 230°C and held for 27 min. Peaks were identified by comparing their retention times with those of authentic reference compounds. The results were expressed as relative area percent of total FAMEs. Because of the high degree of correlation between oleic and linoleic fatty acids (Sabetta et al, 2013; Hernández et al., 2017), both fatty acids were taken in account.

Climate parameters (average temperature and rainfall) were registered under the same period, from April to November, kindly provided by ARSAC, Agrometereology Service. Pearson and Spearman correlation coefficients of the both climate and phenotypic traits among years were calculated using the PAST software (Hammer et al., 2001).

### Population Structure Analysis

SSR analysis was conducted according to Ben Mohamed et al. (2017) using a set of 21 microsatellite markers. A combination of three SSR loci was used in multiplex PCR amplification strategy. DCA3-6Fam, DCA18-6Fam, DCA8-VIC, DCA5-VIC, DCA11-PET, DCA16-6Fam, DCA9-NED (Sefc et al., 2000), GAPU82-NED, GAPU71B-6Fam, (Carriero et al., 2002), UDO4-VIC, UDO12-NED, UDO15-NED (Cipriani et al., 2002) and EMO090-NED (De la Rosa et al., 2002), OLEST1-6Fam, OLEST7-PET, OLEST9-6Fam, OLEST12-6Fam, OLEST14-VIC, OLEST15-VIC, OLEST20-NED, OLEST23-PET (Mariotti et al., 2016) loci were used in this work. PCR products were separated on an ABI PRISM Genetic Analyzer 3130xl (Applied Biosystems Inc., Foster City, CA, USA). Frantoio and Leccino authenticated cultivars were included into the analysis as internal reference to verify the correctness of molecular data. SSR fragments were analyzed by Gene Mapper 3.7 software (Applied Biosystems, USA).

The data obtained by scoring of SSR profiles were used to evaluate the genetic structure of population using STRUCTURE v.2.3.4 (Pritchard et al., 2000) software with K ranging from 1 to 12. The admixture model with correlated allele frequency, a burn-in length of 100.000 followed by 100.000 runs at each K, with three iterations for very K, were used. The true value of K was determined by the Evanno method (Evanno et al., 2005) implemented in Structure Harvester web version 0.6.93 (Earl and Von Holdt, 2012). The Wright’s inbreeding coefficient Fst was calculated using PopGene 1.32.

### Cloning and Sequence Analysis of *FAD2-2* Genomic Clone Including 5′UTR Intron

Genomic DNA from young and healthy leaves collected from the same 97 olive cultivars was prepared using the GenElute™ Plant Genomic DNA Miniprep Kit (Sigma-Aldrich), according to the manufacturer’s protocol. DNA quantification and quality evaluation were carried out by the NanoDrop 2000 spectrophotometer (Thermo Scientific) and samples were then diluted to 10 ng/µl. *Oep*FAD2-2 cDNA sequence isolated from olive by Hernández et al. (2005) was used as template for drawing primer pairs for targeted PCR gene-walking approach to isolate the complete *FAD2-2* gene in the olive cv. Nocellara Messinese. At first, two gene-specific primers (F: 5′-TGAAGGGCGAGCAGTGTGT-3′; R: 5′-CAACTCATTTGATCTTCAACAACCA-3′) were drawn on the 5′ and 3′ terminals of the full-length cDNA sequence, available at the NCBI database (accession n. AY733077.1). These primers amplified the whole genomic region of the gene, which turned out to be much longer than the cDNA sequence; different rounds of nested PCRs followed by direct amplicon sequencing were then performed until the entire genomic sequence was covered. Amplification reactions were performed in a final volume of 20 µl in the presence of 20 ng template DNA, 1× PCR buffer, 1.5 mM of MgCl_2_, 0.5 µM of forward and reverse primers, 0.2 mM of each deoxynucleotide, and 1U Taq DNA polymerase (Invitrogen by Life technologies). Polymerase chain reactions were performed, using a Verity™ Thermal Cycler (Applied Biosystems), as follows: 94°C for 3 min followed by 35 cycles at 94°C for 45 s, 56°C for 30 s, 72°C for 1 min and 30 s, then 72°C for 10 min. PCR products were analyzed on 1.2% agarose gel in 1X TAE. Subsequently, the olive *FAD2-2* gene was sub-cloned into six fragments of approximately 600 bp in PCR-XL-TOPO^®^ vector (Invitrogen by Life technologies) and the recombinant vectors were transformed into competent *E. coli* cells, following the manufacturer’s protocol. The primer list is reported in **Table S2**.

Direct sequencing in both directions of the PCR products was performed on an ABI3130 Genetic Analyzer (Applied Biosystems-Hitachi, United States) using the ABI Prism BigDye Terminator v.3.1. Ready Reaction Cycle Sequencing Kit (Applied Biosystems). An overlapping region on both ends of at least 100 bp from each gene fragment allowed the reconstruction of the entire genomic sequence. The obtained sequences were aligned to the reference cDNA sequence (Hernández et al., 2005) and assembled by SeqMan v.7.0.0 (DNASTAR Lasergene) leading to the two alleles of the gene. In order to confirm the data about homozygous/heterozygous samples for the 117 bp insertion/deletion obtained from the sequence alignment, a gene-specific primer pair (F:5′-CAAGGGATGTTAGGTTGCAG-3′; R:5′-GAGAAATATCAACATCTGTAGGC-3′) was drawn on the sequence fragment containing the insertion/deletion, the DNA of the remaining 96 cultivars was amplified and the corresponding PCR products were analyzed on 1.2% agarose gel in 1X TAE.

In order to evaluate the polymorphisms in the 5′UTR intron, four fragments of about 550 nucleotides length in the intron region of the *FAD2-2* gene were amplified with a set of specific primers (**Table S3**) and sequenced by Sanger method in the 96 olive cultivars selected. Sequence alignment was conducted using the same software above described and SNPs, indels mutations were identified excluding rare SNPs and Indel with a frequency <5%.

The two allelic forms of *FAD2-2* gene of the cv. Nocellara messinese were aligned between them by Clustal Omega Mega-Multiple Sequence Alignment with the Neighbor-joining method (https://www.ebi.ac.uk/Tools/msa/clustalo/10122018), then they were aligned to cv. Farga (Cruz et al., 2016) (https://blast.ncbi.nlm.nih.gov/Blast.cgi/10122018) and var. *sylvestris* (Unver et al., 2017) whole genomes. A publicly available web database, PlantCARE (http://bioinformatics.psb.ugent.be/webtools/plantcare/html/10112018), was used to locate *Cis*-Acting Regulatory Elements in the intron sequence. The intron region of the two allelic forms of *FAD2-2* isolated in *Olea europaea* and in some other plant species (*Sesamum indicum*, *Glycine max*, *Arabidopsis thaliana*, *Brassica napus*, *Perilla frutescens*, *Camelina sativa*, *Carthamus oxyacanthus*, *Carthamus persicus*, *Carthamus tinctorius*, *Salvia hispanica, Sinapis alba*) was analyzed by IMEter v2.0 software, its algorithm is a good predictor of how well the intron sequence will enhance gene expression (Parra et al., 2011).

### Polymorphism, Linkage Disequilibrium Estimation and Single SNP-Based Association Analysis

DnaSp v6. software was used for DNA polymorphism analysis, haplotype reconstruction from unphased data, intragenic recombination (IR) and linkage disequilibrium (LD) degree. For haplotype reconstruction, the algorithm provided by PHASE (Stephens et al., 2001; Stephens and Donnelly, 2003) was used with 1,000 iterations, thinning intervals equal to 10 and 1,000 burn-in iterations. LD between polymorphic sites was estimated by the correlation coefficient (r) calculated from inferred haplotypes. Both the Fischer’s exact test and Chi-square test were used for evaluating significant pairwise associations and Bonferroni correction was also applied. Linkage disequilibrium decay was calculated with the software R 3.4.1 (R Core Team, 2017) by using r^2^ parameters.

Single SNP association analysis was conducted using oleic and linoleic acid content data from 2003 to 2007 years. The mixed linear model (MLM) in Tassel 5.2.51v was implemented with the kinship matrix (K matrix) and the Q matrix, in order to take into account the effects of relatedness among varieties and population structure. The K matrix was calculated using Past software from the 21 SSR markers used for the population structure analysis. Correction for multiple testing was carried out using the estimated false discovery rate (FDR) values (Storey and Tibshirani, 2003) in the R package using function *p.adjust.* Markers with FDR ≤ 0.05 were considered significant. Manhattan plots were visualized using TASSEL 5.2.51v for the single SNP association study. The indels found in the 5′UTR intron were treated as a single polymorphism and computed in association analysis. The TASSEL 5.2.51v software calculates genotypic effect and not allele effect as deviations from the estimated value of the genotypic class with lowest frequency. The class with lowest frequency is set as zero effect, then the other genotype effects are given as deviations between their estimated values and the lowest frequency class.

## Results

### Phenotyping: Fatty Acid Composition Variation

Average rainfall and temperature registered were in a range between 240 (2004) and 658 mm (2005) and 20°C (2005) to 23°C (2003) (**Figure S1**) under the period 2003 to 2007. Highly significant correlations were obtained for temperature among the years with a Pearson’s correlation index ranging from 0.95 to 0.99 while no significant correlations were observed for rainfall among the year except for 2003, 2004 versus 2007 year (**Table 1**), indicating a large rainfall fluctuation over the years. A wide range of variation was observed for the acidic composition (oleic acid: 53–78%; linoleic acid: 3.4–22.5%) covering a large part of the natural variation described for olive (**Table S1**).

**Table 1:** Pearson correlation indexes (A, B) for climate parameters and Spearman correlation indexes (C, D) for fatty acid composition among years. The asterisks indicate the significance of statistical test.

A					C				
Temperature	2003	2004	2005	2006	Oleic acid	2003	2004	2005	2006
2004	0.95***				2004	0.83***			
2005	0.95***	0.95***			2005	0.9***	0.88***		
2006	0.97***	0.98***	0.98***		2006	0.69***	0.65***	0.71***	
2007	0.97***	0.98***	0.98***	0,99***	2007	0.83***	0.88***	0.9***	0.67***
B					D				
Temperature	2003	2004	2005	2006	Oleic acid	2003	2004	2005	2006
2004	−0.04				2004	0.8***			
2005	−0.05	0.04			2005	0.8***	0.9***		
2006	−0.05	0.33***	−0.05		2006	0.6***	0.61***	0.6***	
2007	0.15*	0.07	−0.04	0.07	2007	0.7***	0.6***	0.86***	0.61***

*significant (P = 0.05); ***high significant (P = 0.01).

**Figure 1:**
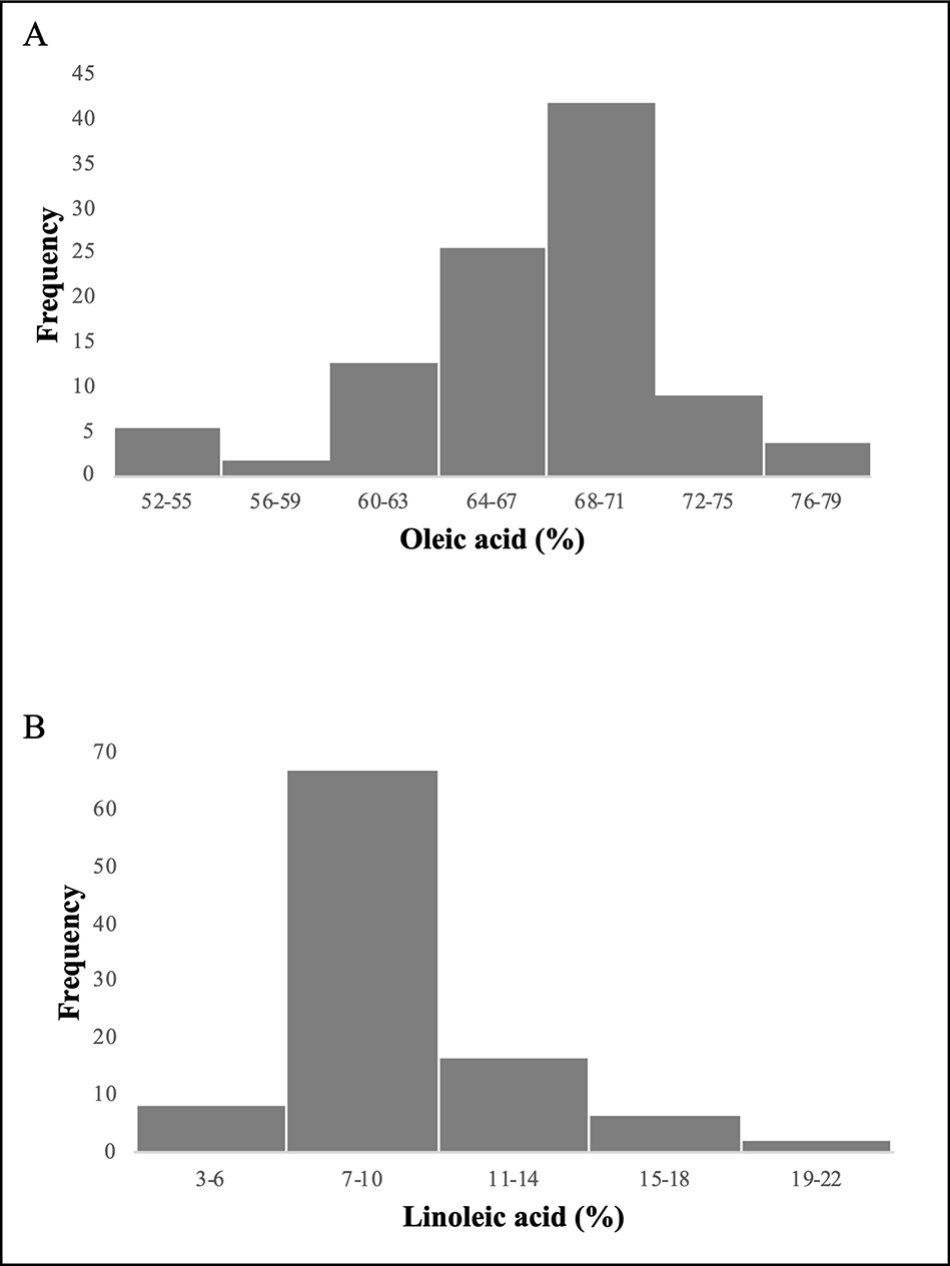
Frequency distribution of the 97 olive varieties for oleic acid **(A)** and linoleic acid **(B)** content.

Since the frequency of phenotypic data showed an asymmetric distribution (**Figure 1**), correlation indexes were calculated using a nonparametric statistical test (Spearman’s correlation index). High significant correlations were observed for both oleic and linoleic acid content among the years at high significance level (P = 0.01). The Spearman’s correlation index ranged from 0.65 to 0.9 and from 0.61 to 0.9 for oleic and linoleic acid, respectively (**Table 1**).

### Population Structure Analysis

The population structure analysis conducted on the 97 olive varieties using a set of 21 SSR markers leaded to 2 main groups, here named ‘Red’ and ‘Green’ (**Figure S2**). A differentiation related to geographic origin was discovered for Sicily and Sardinia cultivars belonging mainly to the red group, while almost all the Abruzzo and Molise cultivars were clustered in the green group. Worthy to note, that almost all the cultivars from Abruzzo clustering in the green group showed a reduced oleic acid content on average (63.64%) in respect of cultivars from Sicilia and Sardinia belonging to the red group showing on average oleic acid content of 68.7% and 68.9% respectively. However, the red and green groups showed a weak difference each other for the oleic acid content, on average 69.3% and 67.5% respectively.

Not a clear differentiation related to geographic origin was highlighted for the other varieties and a lot of admixed genotypes were found. Membership >0.9 was found for the following group of varieties: “Ghiannara,” “Procanica,” “Reale,” “Corsicana da olio,” “Nostrale di Fiano Romano,” “Ottobrina,” “Gaggiolo,” and “Mignolo.” This group of cultivars was considered of clonal origin and excluded from association analysis except one of them (“Mignolo”) considered as reference cultivar.

The Wright’s inbreeding coefficient Fst (Fst = 0.033) confirmed a low degree of population differentiation.

### Genomic Organization, Polymorphisms and Cis-Regulatory Elements of the Olive *FAD2-2* Gene 5′UTR Intron

**Figure 2:**
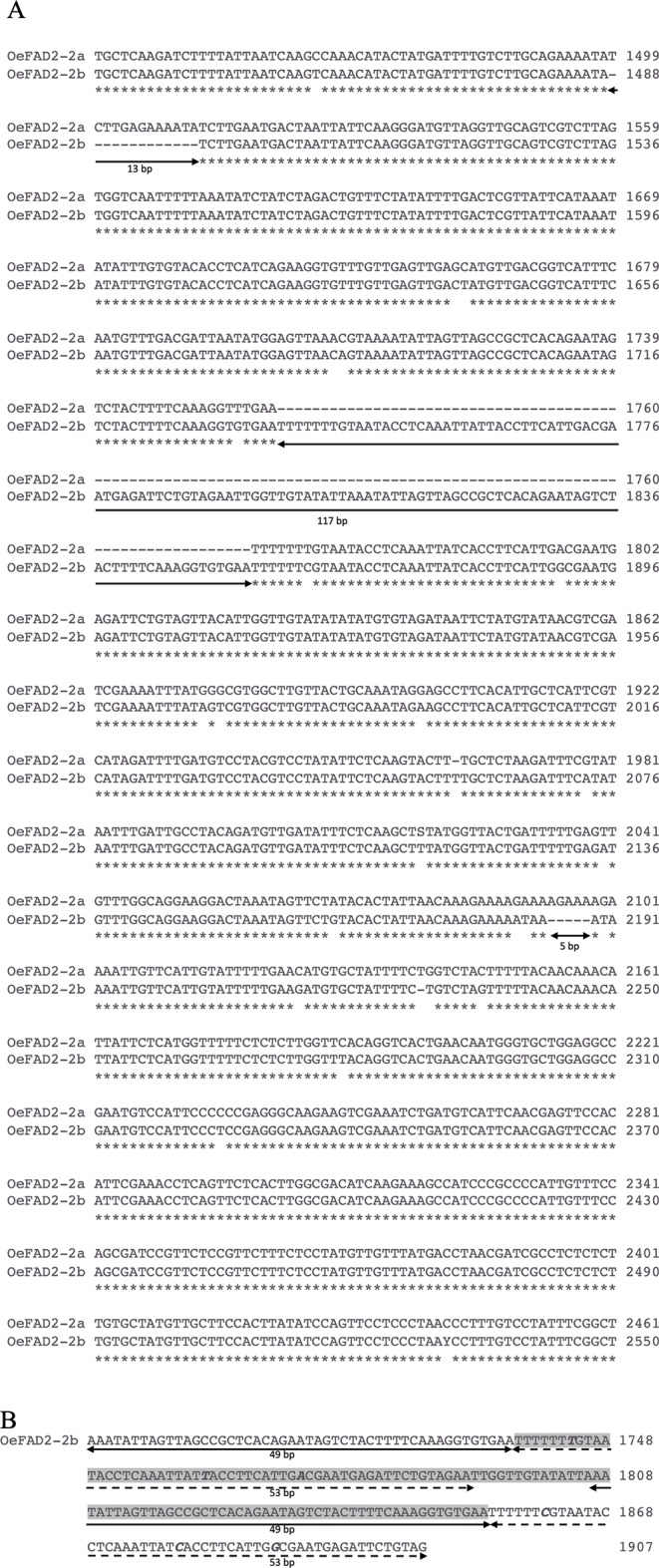
**(A)** Alignment of a fragment of the intron region of *OeFAD2-2a* and *OeFAD2-2b* from cultivar Nocellara messinese. Three long indels of 13 bp, 117 bp and 5 bp, respectively, are underlined. **(B)** Partial view of *OeFAD2-2b* intron region spanning the 117 nucleotide-long insertion (highlighted in grey). Two stretches of 49 nucleotides (underlined with a solid black line) and 53 nucleotides (dashed black line) are duplicated. Polymorphic bases within duplications are marked in bold italic. * Similar nucleotide.

The molecular cloning of *FAD2-2* gene in cv. Nocellara messinese, led to isolate two heterozygous allelic forms, here named *OeFAD2-2a* and *OeFAD2-2b* of 3535 bp and 3624 bp length characterized by 2143- and 2242-bp single introns in the 5′UTR, respectively (**Figure 2A**). Their sequences were deposited in GenBank database *(*Accession numbers MN586855 and MN586856 respectively). The alignments of *OeFAD2-2a* and *OeFAD2-2b* to both wild and cultivated olive whole genomes allowed to locate *OeFAD2-2* on chromosome 17 (Unver et al., 2017) and scaffold Oe6_s00121 (Cruz et al., 2016).

The alignment of the intron regions between the two allele forms revealed three indels: 117, 13, and 5 bp length (**Figure 2A**). The insertion of 117 bp showed two long duplications of 49 and 53 bp (**Figure 2B**) and allowed to distinguish 10, 31, and 45 cultivars in homozygous deletion, homozygous insertion, and heterozygous status, respectively.

Intron sequences showed standard splicing borders GT … AG and were located 11bp from the ATG translation initiation codon. The GC content was 31% indicating a rich component of A+T typically found in other 5′UTR introns (Lozinsky et al., 2014).

**Figure 3:**
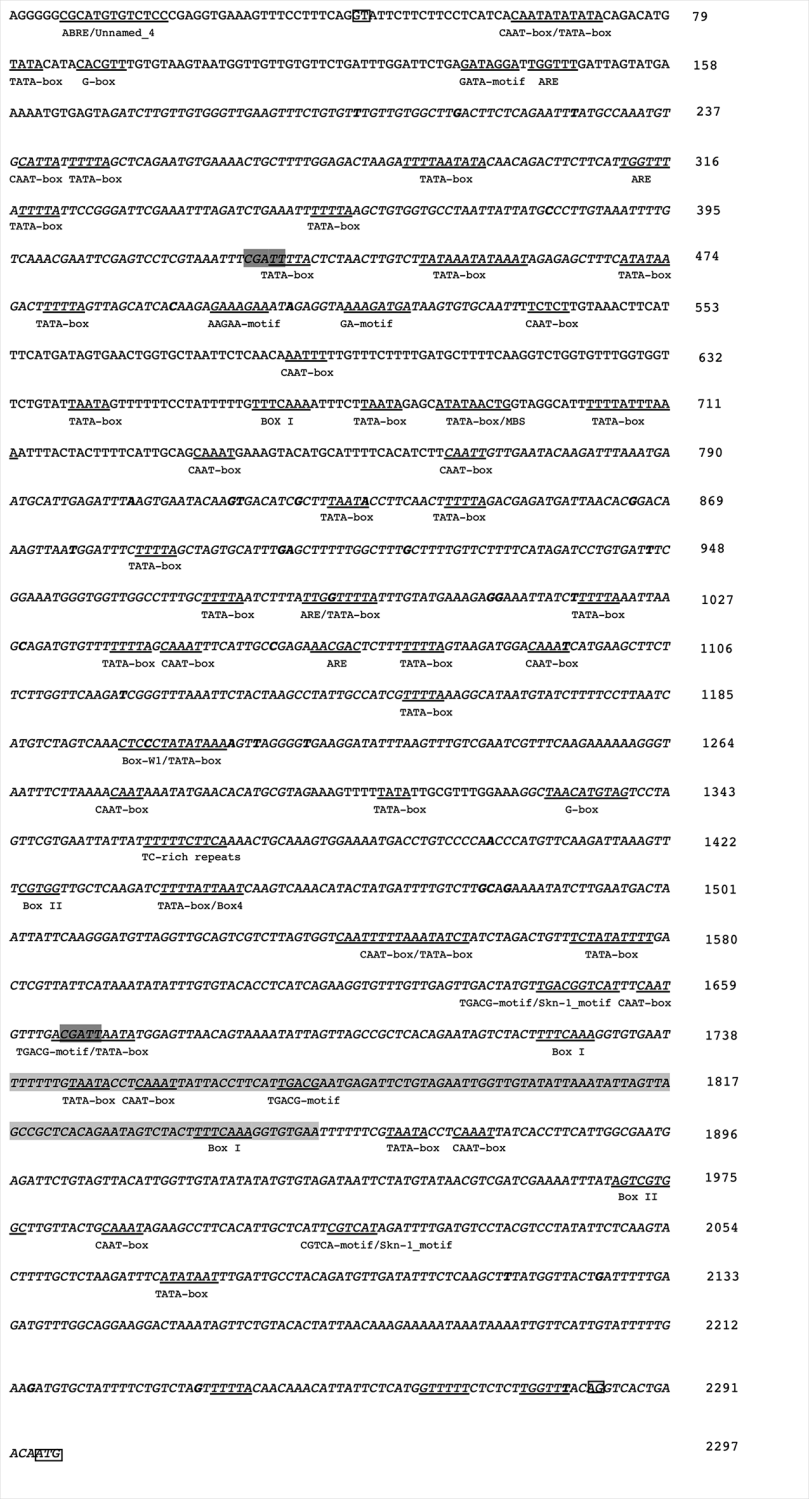
Partial nucleotide sequence of the *OeFAD2-2b* 5′UTR from olive cultivar Nocellara messinese. In italics the sequence regions analyzed in other 96 cultivars and in bold single-nucleotide polymorphisms (SNPs). In the boxes, the GT and AG dinucleotides at both ends of the intronic region and ATG as translational initiation are shown. The insertion of 117bp, not present in the sequence of the *OeFAD2-2a* allele, is shaded grey. In dark grey the pentamer CGATT belonging to IME signals. Moreover, several potential *cis*-regulatory elements are underlined and designated with the names of each of the motifs.

The *OeFAD2-2b* intron sequence was analyzed for known *cis*-acting elements through a web search of publicly available database (PlantCARE). Several *cis*-acting regulatory elements were found (**Figure 3**, **Table S4**), most of them similar to those found in the *SeFAD2* promoter, leading us to speculate a promoter-like role for intron sequence in olive too. The analysis of the intron region of the two allelic forms *OeFAD2-2a* and *OeFAD2-2b* by IMEter v2.0 software revealed a score of 18.11 and 18.24 respectively, higher than *Sesamum indicum* (11.65) and *Brassica napus* (11.76) scores as reported in (**Table S5**). The higher the IMEter score, the more likely the intron is expected to enhance gene expression; in particular, introns that moderately enhance expression tend to have IMEter v2.0 scores above 10 and introns that strongly enhance expression tend to have scores above 20. The pentamer CGATT appears to be an important part of the Intron-Mediated Enhancement (IME) signal, in fact is one of many pentamers used by the IMEter to score introns, and it is the pentamer which shows the biggest difference in frequency between a set of promoter-proximal and promoter-distal introns (Parra et al., 2011). This sequence was detected twice in the 5′UTR intron sequence of *OeFAD2-2*, within TATA box and TGACG-motif/TATAbox, respectively (**Figure 3**).

The SNPs and indel analysis conducted on the 5′UTR intron of the 97 olive cultivars detected 39 SNPs (**Figure 3**). Considering a whole length of the longest of the 5′UTR intron (2242 bp) and excluding indels, a SNP frequency of 1/53 bp was observed. All the selected SNPs were considered common (minor allele frequency > 5%). Among the 39 SNPs individuated, 7 were located within or in close vicinity of *cis*-regulatory elements (**Figure 3**).

### Polymorphism Diversity and Linkage Disequilibrium Estimation

**Figure 4:**
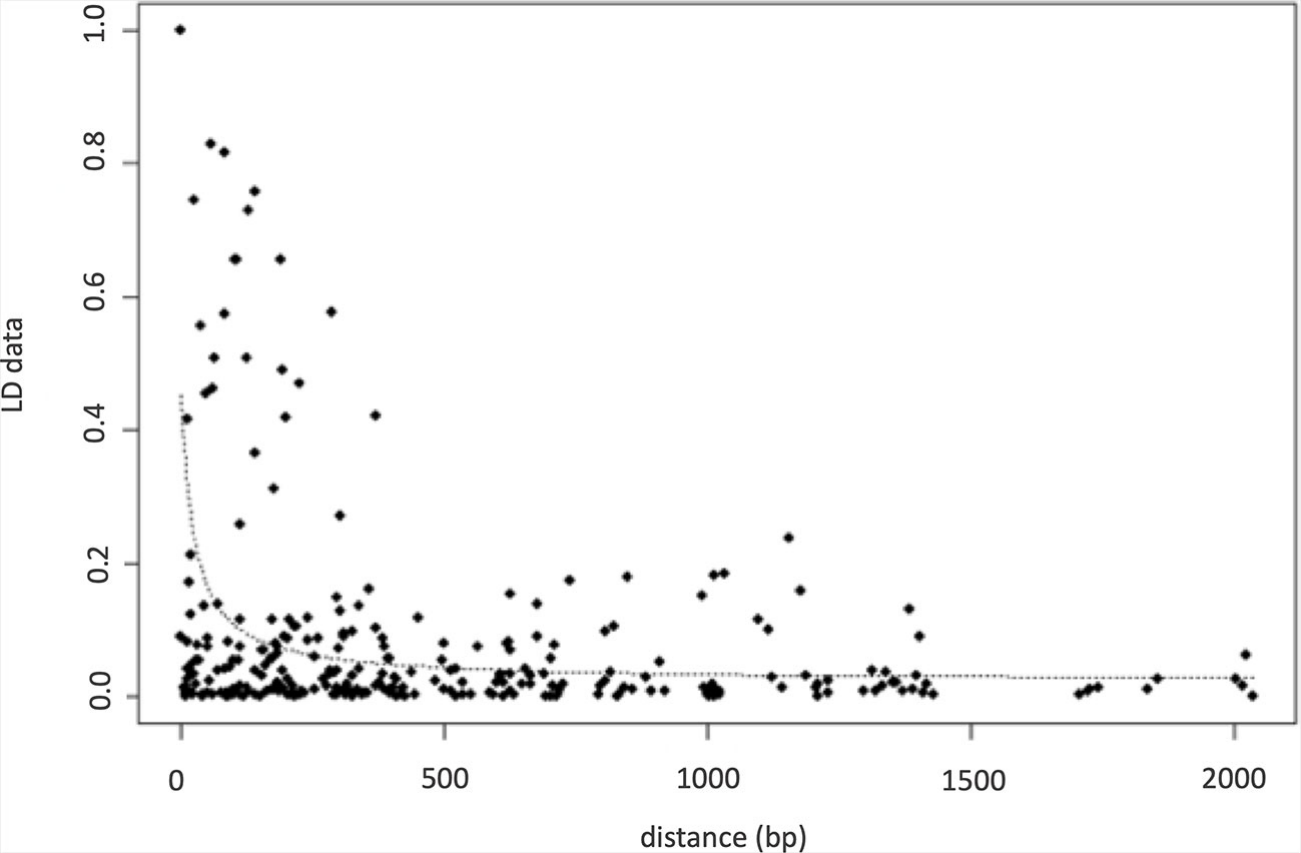
LD decay calculated on inferred haplotypes using r^2^ parameters.

Nucleotide diversity (π) was estimated at 0.0038 indicating a high genetic diversity within the population sample further encouraging the association study. The number of the reconstructed haplotypes by using the DnaSP software was 115. The level of LD between pairs of loci using the inferred haplotypes data of the association population, provided high significant correlations among 16 SNP polymorphisms (**Table 2**) with a range of R from −0.17 to 1. Negative signals indicated a negative correlation between SNPs frequency. The highest positive correlations were found among the following polymorphisms: SNP9, SNP13, SNP14, SNP15, SNP20 with a range of R varying from 0.81 to 1 and between SNP23 and SNP26 with a 0.87 correlation index (**Table 2**). LD decay calculated using inferred haplotypes showed a very quickly decay with a R^2^ dropping to < 0.1 at least 200bp distance within the 5′UTR intron of *FAD2-2* gene (**Figure 4**). The intragenic recombination test confirmed this pattern indicating 174 different recombination events in the 115 calculated haplotypes with 19 minimum number of recombination events. Tajima neutrality test was not statistically significant (D = 0.84) indicating no selection pressure for the 5′UTR intron.

**Table 2:** Results of the LD analysis where the distance between pair of SNPs and their significant pairwise associations were calculated using both the statistical D′ and R.

SNP1	SNP2	Dist	D′*	R*
SNP5	SNP9	324	−1	−0.175
SNP5	SNP13	383	−1	−0.183
SNP4	SNP9	437	−0.833	−0.186
SNP13	SNP167	1338	−0.835	−0.186
SNP14	SNP167	1313	−1	−0.193
SNP15	SNP167	1312	−1	−0.193
SNP5	SNP20	513	−1	−0.195
SNP12	SNP23	194	−1	−0.196
SNP12	SNP26	337	−1	−0.199
SNP4	SNP14	521	−1	−0.201
SNP4	SNP15	522	−1	−0.202
SNP2	SNP12	653	0.696	0.203
SNP2	SNP25	908	0.228	0.227
SNP4	SNP13	496	−1	−0.233
SNP2	SNP4	169	−0.458	−0.236
SNP6	SNP14	394	0.35	0.239
SNP6	SNP15	395	0.35	0.239
SNP12	SNP25	255	−1	−0.241
SNP2	SNP168	2023	0.636	0.245
SNP14	SNP23	157	−1	−0.263
SNP15	SNP23	156	−1	−0.264
SNP14	SNP26	300	−1	−0.266
SNP15	SNP26	299	−1	−0.267
SNP5	SNP23	565	−0.816	−0.27
SNP4	SNP5	113	0.349	0.273
SNP13	SNP23	182	−0.894	−0.273
SNP5	SNP26	708	−0.819	−0.274
SNP11	SNP12	32	1	0.277
SNP6	SNP20	499	0.333	0.281
SNP2	SNP11	621	−0.329	−0.284
SNP9	SNP23	241	−1	−0.291
SNP20	SNP23	52	−0.905	−0.294
SNP9	SNP26	384	−1	−0.295
SNP11	SNP168	1402	−0.895	−0.298
SNP20	SNP26	195	−0.906	−0.298
SNP6	SNP9	310	0.404	0.306
SNP13	SNP26	325	−1	−0.309
SNP25	SNP168	1115	0.817	0.313
SNP6	SNP13	369	0.401	0.318
SNP4	SNP26	821	−0.754	−0.323
SNP14	SNP25	218	−1	−0.323
SNP15	SNP25	217	−1	−0.324
SNP25	SNP167	1095	0.564	0.337
SNP20	SNP25	113	−0.851	−0.339
SNP11	SNP20	174	0.741	0.34
SNP13	SNP25	243	−0.917	−0.343
SNP4	SNP11	452	0.576	0.344
SNP167	SNP168	20	0.546	0.351
SNP9	SNP25	302	−1	−0.357
SNP11	SNP167	1382	−0.7	−0.362
SNP5	SNP11	339	0.781	0.366
SNP11	SNP13	44	0.856	0.369
SNP4	SNP23	678	−0.875	−0.37
SNP11	SNP15	70	1	0.371
SNP11	SNP14	69	1	0.372
SNP2	SNP6	296	0.673	0.385
SNP2	SNP26	990	0.466	0.387
SNP5	SNP25	626	−0.964	−0.392
SNP23	SNP168	1176	0.845	0.397
SNP6	SNP12	357	0.789	0.402
SNP9	SNP11	15	1	0.412
SNP4	SNP25	739	−0.804	−0.417
SNP2	SNP23	847	0.513	0.421
SNP26	SNP167	1013	0.59	0.426
SNP26	SNP168	1033	0.922	0.428
SNP23	SNP167	1156	0.667	0.488
SNP12	SNP20	142	1	0.604
SNP12	SNP13	12	1	0.644
SNP11	SNP26	369	−0.905	−0.648
SNP9	SNP12	47	1	0.674
SNP23	SNP25	61	0.832	0.679
SNP11	SNP23	226	−0.968	−0.685
SNP12	SNP14	37	1	0.746
SNP12	SNP15	38	1	0.746
SNP25	SNP26	82	0.917	0.757
SNP11	SNP25	287	−0.874	−0.759
SNP9	SNP20	189	0.903	0.81
SNP14	SNP20	105	1	0.81
SNP15	SNP20	104	1	0.81
SNP13	SNP20	130	0.91	0.854
SNP13	SNP14	25	1	0.863
SNP13	SNP15	26	1	0.863
SNP23	SNP26	143	0.88	0.87
SNP9	SNP14	84	1	0.903
SNP9	SNP15	85	1	0.903
SNP9	SNP13	59	0.952	0.91
SNP14	SNP15	1	1	1

*Significant pairwise associations using both the Fischer’s exact test and Chi-square test and Bonferroni correction.

### Trait-Marker Association Analysis

**Figure 5:**
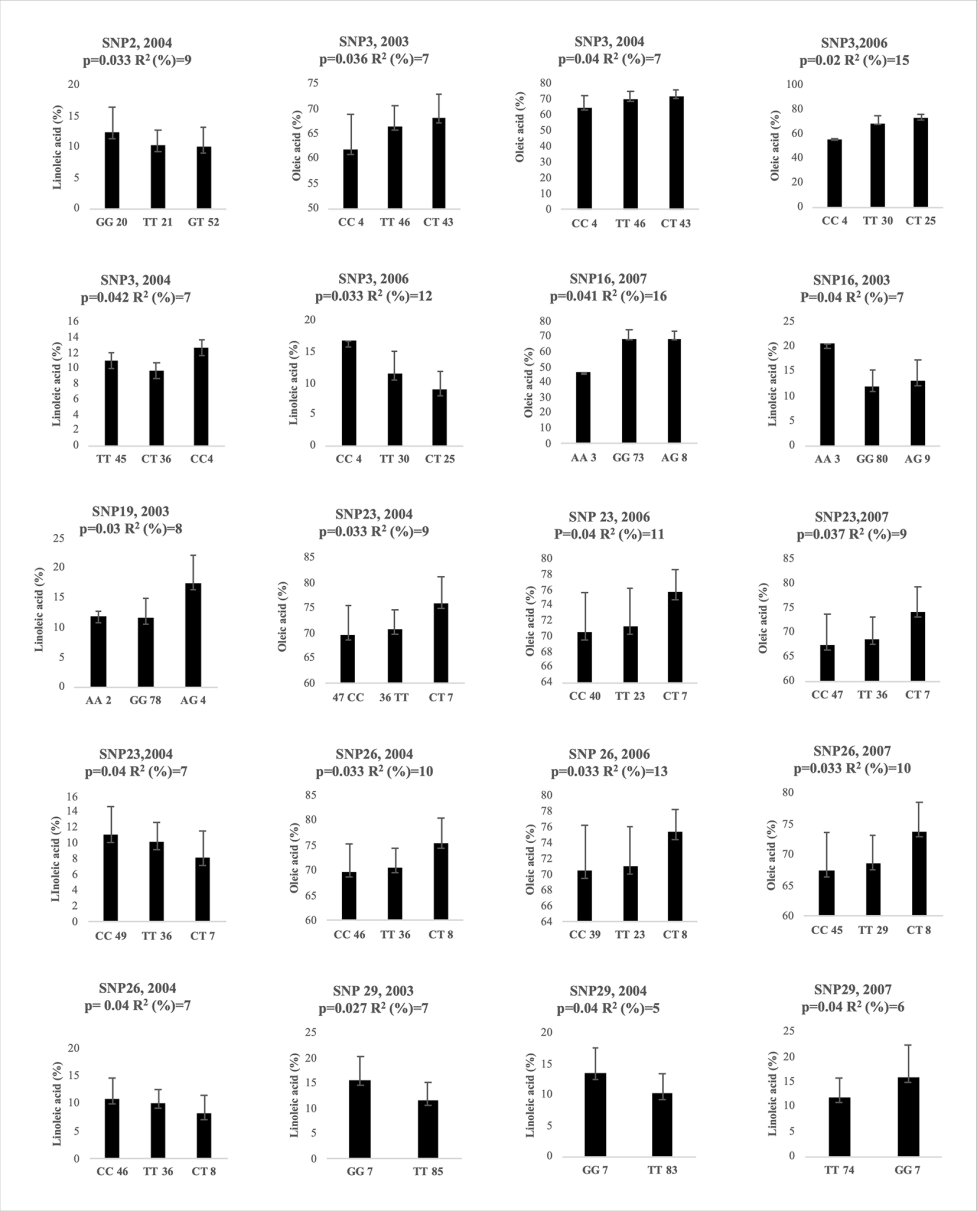
Genotypic effects of the significantly associated SNPs on oleic and linoleic acid content in different years. The X-axis indicates the genotype status of cultivars (letters) and the absolute frequency of genotypes (number). R^2:^ is the statistical used for association analysis and p is the Benjamini-Hochberg Adjusted p value.

The association analysis, carried out between 39 SNPs and oleic and linoleic acid content for 4 years, using the mixed linear model (MLM) with Q matrix and kinship included, allowed to individuate 20 significant associations (P< 0.05) after correction for multiple testing, for 7 SNPs (**Figure 5**). The SNP3, SNP23, SNP26 and SNP29 resulted significantly associated in three years, the SNP16 in two years, while the SNP2 and the SNP19 were significant only for one year (**Figure 5**). Among the indels analyzed, only the 13bp indel was significantly associated to both oleic and linoleic acid but only in 2006 year (data not shown). Marked differences in oleic acid content were observed between homozygous and heterozygous genotypes for SNP3, SNP23, and SNP26 (**Figure 5**) for all three years where they resulted significantly associated. This pattern of gene action suggested an over- or under dominance effects. Homozygous genotypes decreased oleic acid content with the same pattern for all three years with a negative effect of −3 and −10 for TT and CC genotypes, respectively, versus the heterozygous genotype in the SNP3. Similar values of genotype effects were observed for both the SNP23 and SNP26, with −5 and −6 values for the CC and TT homozygous genotypes. Less marked differences were observed for linoleic acid content (**Figure 5**), probably due to the minor range of variation. Interestingly, genetic population structure analysis clustered almost all the Abruzzo cultivars in a single group showing the CC/TT homozygous genotype for both the SNP23 and SNP26 except the cultivar Dritta showing heterozygous genotype for the latter SNP.

The SNP26 was located within the joint elements box-W1/TATA box (**Figure 3**). The SNP2 resulted significantly associated only to the linoleic acid in 2004 with a gene action pattern consistent with an additive effect. The SNP16 seemed to show a similar pattern too. The SNP19 had a pattern probably consistent with an over dominance effect considering the great increment of linoleic acid by heterozygous genotype in respect of that of two homozygous one. The indel of 13bp resulted significantly associated after multiple testing correction (data not shown) for 10 individual polymorphisms contributing to explain 13% and 9% phenotypic variance for the oleic and linoleic acid content, respectively. The proportion of phenotypic variation explained by the associated SNPs and indel varied among the years ranging from 7% to 16% (**Figure 5**). On average SNP3, SNP23, SNP26 explained the major phenotypic variance with 9.7%, 9.6% and 11% for oleic acid content.

## Discussion

In this work, starting from the cDNA of the *FAD2-2* gene isolated from Hernández et al. (2005), a complete genomic clone was isolated by a gene-walking approach and four fragments of the 5′UTR intron were characterized through an *in silico* and structural analysis with the aim to explore the natural allelic variability of *FAD2-2* in 97 olive varieties and its role in the gene expression regulation. The molecular cloning of *FAD2-2* gene allowed to distinguish two allelic forms, *OeFAD2-2a* and *OeFAD2-2b*. A single intron in the 5′UTR was isolated, and three indels were individuated. In particular, the insertion of 117 bp showed very interestingly two long duplications of 49 and 53 bp. No duplications have been previously individuated in the 5′UTR intron of *FAD2-2* genes in olive. Similarly, Cultrera et al. (2019) analyzing polymorphisms of different gene fragments belonging to crucial metabolic pathways, found a tandem duplication made up of a 166 bp motif within *OeSUT1* exon in olive. Zeng et al. (2017) found a transposable element insertion at position −26 bp in the 5′ upstream region from the translation start codon in *FAD2* gene in *Sinapis alba*. Martínez-Rivas et al. (2001) and Cao et al. (2013) asserted the *FAD2* genes family evolved by duplication from constitutive expressed *FAD2* genes, and recently, it was confirmed in wild olive (Unver et al., 2017).

In this work, the hypothetical mechanisms concerning the origin and evolution of introns have not been explored, but the presence of two duplications within the 5′UTR intron led us to speculate other mechanisms could be occurred in the differentiation of *FAD2* genes, such as the multiplication of a preexisting intron by tandem duplication or creation of a new intron by internal gene duplication (Gao and Lynch, 2009; Ma et al., 2016).

It is known that the presence of a 5′UTR intron can enhance gene expression depending on different characteristics of the intron: i) different size of the intron; ii) distribution of the motifs dispersed throughout the 5′ intron region iii) position of intron with respect to the 5′UTR and the translation start site (Chung et al., 2006). The 5′UTR lengths vary dramatically among individual genes in higher eukaryotes and can range from a few to thousands of base pairs. This large range of 5′UTR lengths suggests that there may be greater regulation of specific mRNA subsets (Leppek et al., 2018). Without any doubt, the duplication event here found, increased the size of the intron in the 5′UTR of the *FAD2-2* gene in olive.

The IMEter score here found for *OeFAD2-2a* and *OeFAD2-2b* introns indicated a medium-high induction of the gene expression. Genes with the most powerful IME signals appear to be highly and widely expressed housekeeping genes (Parra et al., 2011). A phylogenetic analysis of FAD2 and FAD6 enzymes conducted by Hernández et al. (2005) led to classify *OeFAD2-2* gene as housekeeping-type. Expression analysis of olive *FAD2-2* gene showed that it is highly expressed in mesocarp and seed during the ripening period of olive fruit (Hernández et al., 2009). Different authors reported a constitutively expression of *FAD2-2* genes but with a differentiated spatial and temporal expression level regulation as well (Jin et al., 2001; Zhang et al., 2009; Dar et al., 2017). *FAD2* genes seem to play a key role for some crucial processes for the plant survival such as fatty acid synthesis, plant development, cold and salt tolerance (Dar et al., 2017). In olive, *FAD2-2* gene was shown to be the main gene responsible for the oleic acid desaturation with a differentiated gene expression during the ripening stages well correlated with linoleic acid biosynthesis pattern (Hernández et al., 2009). Furthermore it seems involved in cold tolerance (Matteucci et al., 2011). It was also shown in olive a different expression level between two olive cultivars, Picual and Arbequina, induced by low and high temperature, darkness, and wounding, without changing the oleic and linoleic acid contents in the mesocarp (Hernández et al., 2011). In addition, in Arbequina cultivar, *FAD2-2* is involved in the response to draught (Hernández et al., 2009). Expression levels of olive *FAD2* genes have also been studied in relation to regulated deficit irrigation and salt stress (Hernández et al., 2018; Moretti et al., 2019).”

The *in silico* analysis of the 5′UTR intron in the *FAD2-2* gene showed *cis*-acting elements putatively involved in above described responses. Additional *cis*-acting elements found in the duplications such as TATA box and CAAT box; TGACG-motif and the Box 1 involved to abscisic acid (ABA) and light response, respectively were found and seem to indicate an evolutionary pathway toward an enhancing of the expression level rather than new functionalization. In fact, it could also have to do with the evolutionary option aiming to maintain high the energetic and time costs to transcribe and splice introns, option that could be significant enough to influence the organism’s phenotype. For instance, some highly expressed genes are found under strong selection to remain intron-poor for transcriptional efficiency, whereas other genes are found to have longer and numerous introns to enhance expression (Lozada-Chávez et al., 2018). No relationships were found when the 117 bp insertion was analyzed for significant associations with the acidic content variation, but other biological processes, here not studied, could be involved.

A double presence of the pentamer CGATT as part of IME signals, the very near location of the intron (11bp) to the translational starting site and the duplications within the sequence probably could contribute overall to enhance the gene expression level (Chung et al., 2006; Lozinsky et al., 2014).

The SNP frequency detected in the 5′UTR intron was lower than those found in intron region of genes belonging to the same primary biosynthetic pathway such as acyl carrier protein (ACP) genes (Cultrera et al., 2019), even if this author found on average a higher SNP frequency than other species. Although in this study the neutrality test was not significant, it is worthy to note that 5′UTR introns may be subject to different selective forces from the introns in CDSs and 3′UTRs, possibly due to a specific regulatory role in gene expression (Chung et al., 2006). For instance, differences in the rate of evolution of *FAD2* 5′UTR were found in *Gossypium* species (Liu et al., 2001) suggesting that the selection pressure on these regions could be really different.

Although the Wright’s inbreeding coefficient indicated a low degree of population differentiation in general confirmed also by a weak difference in oleic acid content between red and green group, interesting correlations were observed among almost all the Abruzzo cultivars, (clustering in the green group), their allelic homozygous status for the both SNP23 and SNP26 and oleic acid content. Moreover, the same average oleic acid content found in Sicilian and Sardinian cultivars clustering in the same group, suggested a strong genetic relationship as found also by other authors (Baldoni et al., 2006). Similarly, other authors found a correlation between phenotypic traits and genetic population structure in olive (D’Agostino et al., 2018; Zhu et al., 2019) confirming a high heritability of the analyzed traits (D’Agostino et al., 2018).

The correlation between alleles in a population is stated by LD (Myles et al., 2009). The pattern and extent of LD determines the resolution of association mapping studies (Flint-Garcia et al., 2003). For outcrossing species like most trees, rapid LD decay was reported (Krutovsky and Neale, 2005; Ingvarsson, 2005; Wegrzyn et al., 2010) and for these species, a large number of markers are required to detect significant marker-trait associations (Flint-Garcia et al., 2003; Myles et al., 2009). In fact, a high number of recombination events have been here found and very quick LD decay has been observed, but if the physical position of mutations was known, probably a slower LD decay would had expected as observed by Cultrera et al. (2019).

The association study allowed to individuate 7 SNPs significantly associated to the oleic and linoleic content variation. Some of these associations were confirmed along the years although rainfall fluctuations were observed. These results confirmed the high heritability of fatty acid composition (Ripa et al., 2008; Dabbou et al., 2010; De la Rosa et al., 2016). However, a low number of genotypes associated with a few SNPs (SNP3, SNP16 and SNP19) for the trait “low oleic/high linoleic content” were observed due to a sampling bias of the population that explains in fact the asymmetric distribution of the frequency classes for oleic and linoleic acid traits. This pattern of distribution of phenotypic variation will need to be enlarged in the future studies.

All the SNPs significantly associated, were located near or outside of the *cis*-acting elements putatively involved in fatty acid biosynthesis regulation. The 5′ and 3′ untranslated regions (UTRs) are non-coding and do not directly contribute to the protein sequence. Free from the constraints of encoding proteins, UTRs can form considerable Watson–Crick and non-canonical base pairing that can potentially impact every step of translation (Leppek et al., 2018). Despite the evolutive conservation of the 5′UTR intron, the high structural variability found among and within the species makes difficult to speculate about a specific regulation mechanism (Lozinsky et al., 2014).

All the associations identified in this study explained a small proportion of the phenotypic variance. These small effects attributed to individual SNPs were consistent with earlier studies in accordance with polygenic quantitative models of plant traits (Eckert et al., 2009; Tian et al., 2014).

Although a higher number of genotypes probably are needed in olive, two SNPs in high LD seem to give a contribute to the oleic acid increasing/linoleic acid reduction in a genotypic way referring to a under/over-dominance effect of the heterozygous CT genotypes. These results are consistent with the high heterozygous status of the olive genome (Muleo et al., 2016) and led us to speculate that acidic composition variation within *Olea europaea* L. species might be regulated by mutations within the *FAD2-2* 5′UTR intron.

In conclusion, our work confirmed the presence of a large intron within the 5′UTR of the *FAD2-2* gene also in the olive tree, highlighting the presence of a double duplication. The *in silico* analysis addressed us toward a putative role of the 5′UTR intron in the regulation of gene expression showing several *cis*-regulatory elements. Furthermore, the LD and association analysis showed that the SNP23 and SNP26 resulted strictly associated each other and seemed to contribute to the increase of oleic acid/reduction of linoleic acid. These results will be validated by an analysis of gene expression in order to confirm the putative regulation mechanisms here raised.

## Data Availability Statement

The sequencing data has been deposited in GenBank and can be found using the following accession numbers: Oe-FAD2-2a (BankIt2272927 Seq1 MN586855) and Oe-FAD2-2b (BankIt2272927 Seq2 MN586856).

## Author Contributions

SZ designed, wrote the manuscript, statistical analysis about phenotyping, genetic population structure, LD and association mapping study. AS and SM isolated FAD2-2 full gene, 5′UTR intron. AS conducted SSR genotyping and all the bioinformatic analysis. FC and AT helped for bioinformatic and statistical analysis. FL conducted running of SSR fragments to the genetic sequencer. CB, ER, MP, and EP conducted phenotyping for chemical composition of olive oil. AI conducted statistical analysis using R software package.

## Conflict of Interest

The authors declare that the research was conducted in the absence of any commercial or financial relationships that could be construed as a potential conflict of interest.
