# Supplementary figures and images for: Association Study of the 5′UTR Intron of the FAD2-2 Gene With Oleic and Linoleic Acid Content in Olea europaea L
Source: Front Plant Sci. 2020 Feb 13;11:66. doi: 10.3389/fpls.2020.00066 (PMC7031445; doi:10.3389/fpls.2020.00066)

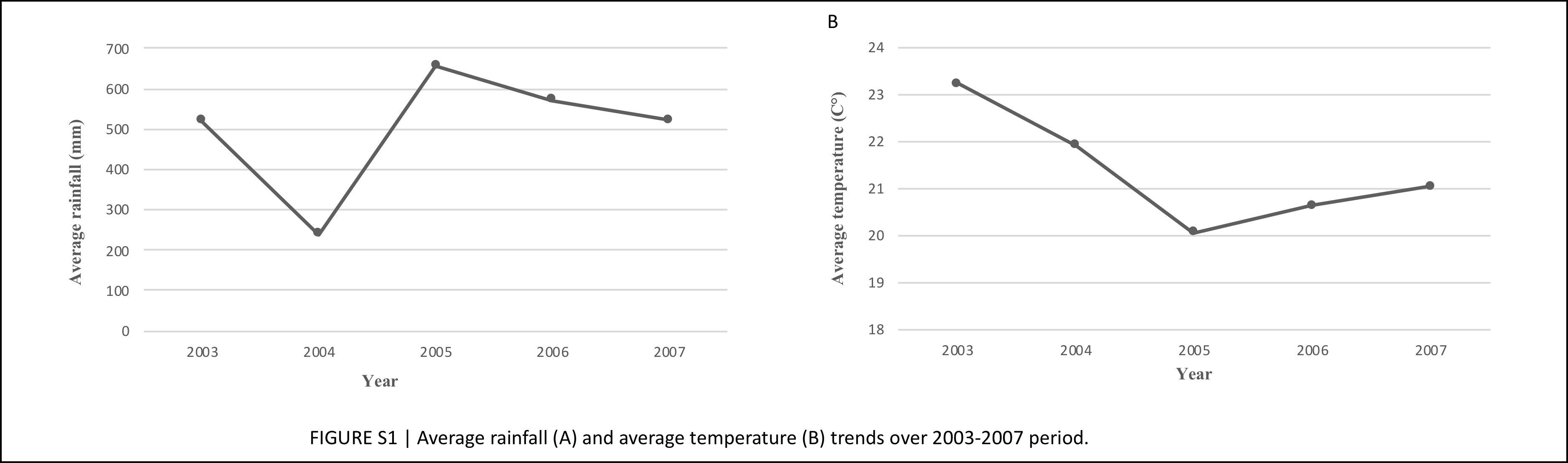

Supplement: Supplementary file 1 [file Image_1.tiff]

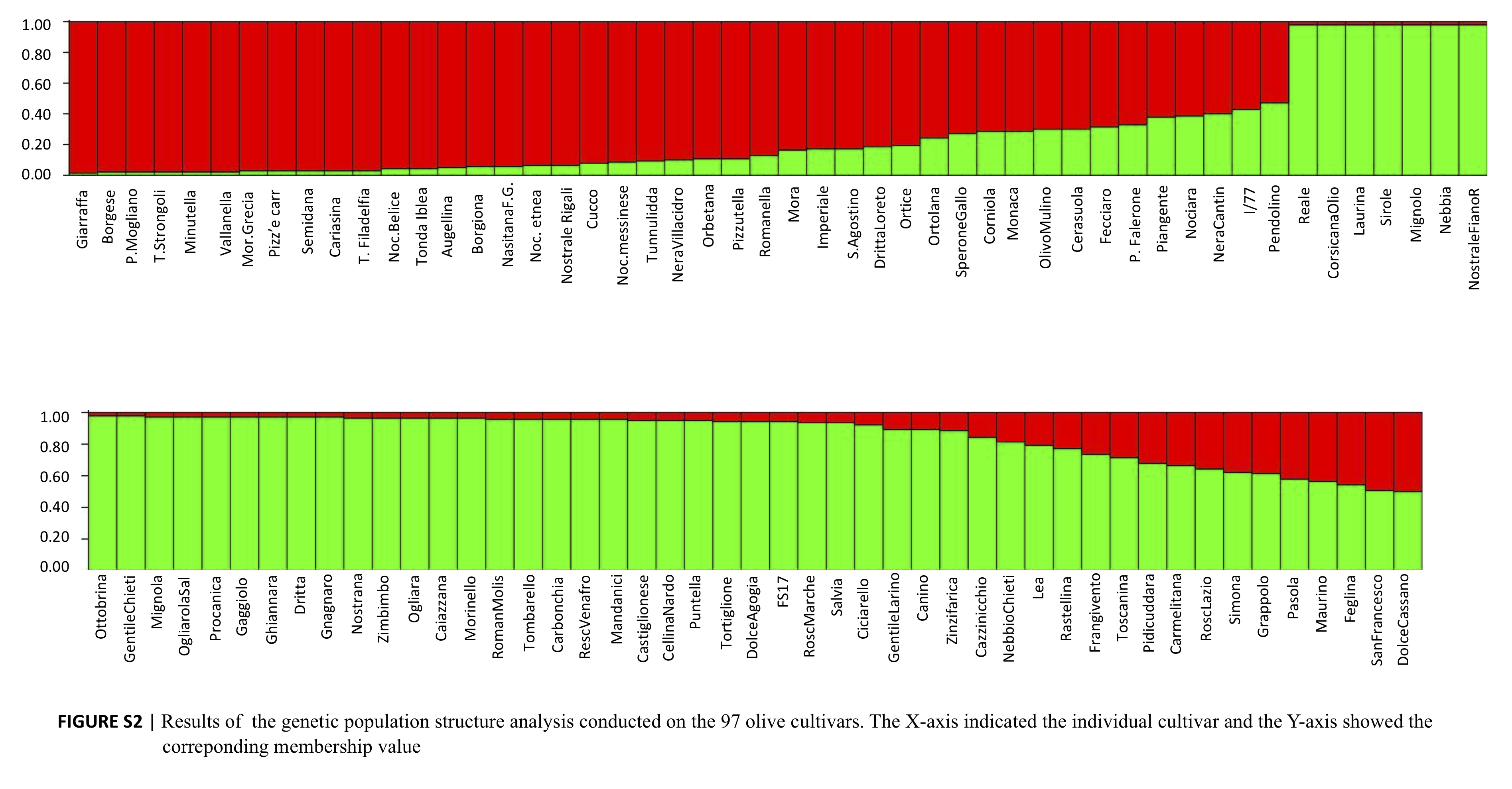

Supplement: Supplementary file 2 [file Image_2.tiff]
