# Supplementary material for: Association Study of the 5′UTR Intron of the FAD2-2 Gene With Oleic and Linoleic Acid Content in Olea europaea L
Source: Front Plant Sci. 2020 Feb 13;11:66. doi: 10.3389/fpls.2020.00066 (PMC7031445; doi:10.3389/fpls.2020.00066)
Supplement: Supplementary file 3 [file Table_1.docx]

**TABLE S1** | List of the olive varieties investigated, their provenience, average content with standard deviation of oleic and linoleic acid.

| **Cultivar Code** | **Cultivar** | **Provenience** | **Average Oleic**  **Acid Content (%)** | **Average Linoleic Acid Content %)** |
| --- | --- | --- | --- | --- |
| 10 | Augellina | Basilicata | 72.9±0.95 | 10.4±0.91 |
| 18 | Borgese | Calabria | 78.3±1.41 | 7.8±0.81 |
| 180 | Borgiona | Umbria | 71.8±6.82 | 9.7±3.72 |
| 240 | Caiazzana | Campania | 55.9±2.46 | 19.9±2.45 |
| 32 | Canino | Lazio | 72.7±3.11 | 8.7±0.41 |
| 216 | Carbonchia | Abruzzo | 53.2±41 | 22.5±3.11 |
| 72 | Cariasina | Sardegna | 70.9±4.82 | 11.1±3.43 |
| 120 | Carmelitana | Puglia | 68.4±4.43 | 11.8±1.82 |
| 1 | Castiglionese | Abruzzo | 66.9±4.23 | 11.6±1.96 |
| 121 | Cazzinicchio | Puglia | 69.2±2.51 | 10.5±1.76 |
| 55 | Cellina di Nardo' | Puglia | 75.4±1.01 | 10.5±1.32 |
| 80 | Cerasuola | Sicilia | 76.3±1.71 | 6.1±0.81 |
| 115 | Ciciarello | Calabria | 55.3±1.42 | 16.9±1.52 |
| 192 | Corniola | Calabria | 71.6±1.25 | 9.3±0.23 |
| 73 | Corsicana da olio | Sardegna | 71.8±1.52 | 9.4±2.31 |
| 3 | Cucco | Abruzzo | 72±1.31 | 7.4±0.62 |
| 97 | Dolce Agogia | Umbria | 73±2.61 | 14.1±2.08 |
| 122 | Dolce di Cassano | Puglia | 72.3±1.82 | 9.2±1.05 |
| 4 | Dritta | Abruzzo | 62.9±0.24 | 15.9±0.68 |
| 232 | Dritta di Loreto | Marche | 70.7±3.85 | 10.7±3.27 |
| 154 | F.S.17 | Umbria | 70.6±3.52 | 9.9±2.11 |
| 171 | Fecciaro | Umbria | 68.8±2.71 | 8.9±3.21 |
| 47 | Feglina | Liguria | 70.5±3.71 | 13.8±3.19 |
| 58 | Frangivento | Puglia | 72.2±2.54 | 8.8±1.64 |
| 52 | Gaggiolo | Lombardia | 72.4±2.95 | 8.8±2.25 |
| 5 | Gentile di Chieti | Abruzzo | 63.2±4.11 | 14.6±2.11 |
| 166 | Gentile di Larino | Molise | 70.7±2.51 | 10.4±0.81 |
| 263 | Ghiannara | Basilicata | 69.1±3.43 | 11.2±1.91 |
| 81 | Giarraffa | Sicilia | 64.9±3.45 | 16.9±3.19 |
| 187 | Gnagnaro | Molise | 64.9± 2.92 | 15.4±1.95 |
| 238 | Grappolo | Toscana | 76.4± 3.46 | 8.1±2.25 |
| 179 | I/77 | Umbria | 75.5±3.44 | 8.6±1.55 |
| 158 | Imperiale | Calabria | 69.8±1.48 | 12.1±2.37 |
| 151 | Laurina | Marche | 61.1±0.91 | 14.5±0.76 |
| 104 | Lea | Marche | 69.1±2.34 | 9.6±0.96 |
| 125 | Mandanici | Sicilia | 71.4±1.65 | 6.8±0.51 |
| 135 | Maurino | Toscana | 70.4±6.01 | 10.1±2.43 |
| 54 | Mignola | Marche | 68.1±0.46 | 9.7±2.02 |
| 136 | Mignolo | Toscana | 71.1±2.68 | 9.7±1.42 |
| 36 | Minutella | Lazio | 66.4±1.48 | 12.3±0.56 |
| 212 | Monaca | Sicilia | 62.1±2.54 | 14.2±0.90 |
| 131 | Mora | Puglia | 57.1±7.91 | 20.4±5.60 |
| 178 | Morellona di Grecia | Puglia | 71.6±1.11 | 8.6±0.47 |
| 239 | Morinello | Toscana | 67.7±2.87 | 11.1±0.55 |
| 195 | Nasitana frutto grosso | Sicilia | 63.3±1.91 | 12.6±0.88 |
| 105 | Nebbia | Marche | 68.1±2.12 | 10.9±1.18 |
| 102 | Nebbio di Chieti | Abruzzo | 63.2±4.41 | 15.6±3.04 |
| 23 | Nera di Cantinelle | Calabria | 72.6±2.72 | 9.5±1.84 |
| 113 | Nera di Villacidro | Sardegna | 70.5±2.02 | 11.1±1.78 |
| 85 | Nocellara del Belice | Sicilia | 70.2±1.24 | 10.9±0.86 |
| 86 | Nocellara Etnea | Sicilia | 68.1±2.15 | 12.9±2.49 |
| 87 | Nocellara messinese | Sicilia | 63.4±1.65 | 16.6±1.42 |
| 61 | Nociara | Puglia | 70.9±1.38 | 9.9±0.94 |
| 37 | Nostrale di Fiano Romano | Lazio | 71.6±0.93 | 11.1±1.46 |
| 98 | Nostrale di Rigali | Umbria | 66.8±4.96 | 12.3±2.92 |
| 24 | Nostrana | Calabria | 66.6±1.82 | 14.8±1.70 |
| 245 | Ogliara | Campania | 68.7±2.51 | 11.1±1.37 |
| 64 | Ogliarola Salentina | Puglia | 67.4±3.78 | 11.1±1.82 |
| 140 | Olivo del mulino | Toscana | 74.5±2.13 | 6.37±0.54 |
| 107 | Orbetana | Marche | 55.4±5.65 | 20.35±2.98 |
| 201 | Ortice | Campania | 72.6±2.52 | 8.72±0.73 |
| 161 | Ortolana | Campania | 72.8±1.65 | 11.13±2.22 |
| 48 | Ottobrina | Liguria | 67.1±2.37 | 11.81±1.26 |
| 65 | Pasola | Puglia | 69.8±2.21 | 14.68±2.35 |
| 95 | Pendolino | Toscana | 72.8±1.82 | 8.64±1.06 |
| 138 | Piangente | Toscana | 73.1±4.54 | 9.57±2.82 |
| 106 | Piantone di Falerone | Marche | 71.7±1.26 | 10.11±1.85 |
| 108 | Piantone di Mogliano | Marche | 73.8±3.76 | 9.1±2.90 |
| 153 | Pidicuddara | Sicilia | 72.5±3.67 | 9.7±1.55 |
| 75 | Pizz'e Carroga | Sardegna | 63.9±3.81 | 13.83±2.60 |
| 213 | Pizzutella | Sicilia | 67.6±1.25 | 11.41±0.96 |
| 38 | Procanica | Lazio | 69.9±1.28 | 8.15±5.86 |
| 177 | Puntella | Abruzzo | 57.7±2.63 | 15.84±1.04 |
| 172 | Rastellina | Umbria | 64.6±2.88 | 8.75±1.05 |
| 39 | Reale | Lazio | 71.3±1.97 | 9.95±1.27 |
| 189 | Resciola di Venafro | Molise | 68.6±0.74 | 10.83±0.54 |
| 129 | Romanella | Calabria | 66.2±2.44 | 12.13±2.46 |
| 167 | Romanella Molisana | Molise | 72.2±2.53 | 7.42±2.14 |
| 41 | Rosciola del Lazio | Lazio | 62.8±2.25 | 16.43±2.19 |
| 110 | Rosciola Marche | Marche | 64.6±3.98 | 15.14±1.87 |
| 67 | Sant' Agostino | Puglia | 74.8±2.93 | 8.56±2.65 |
| 144 | San Francesco | Toscana | 76.9±2.01 | 5.8±0.66 |
| 42 | Salvia | Lazio | 72.1±1.71 | 10.68±1.00 |
| 76 | Semidana | Sardegna | 67.4±3.81 | 12.78±1.65 |
| 68 | Simona | Puglia | 78.4±1.12 | 3.59±0.65 |
| 44 | Sirole | Lazio | 68±1.51 | 11.33±0.61 |
| 175 | Sperone di Gallo | Molise | 60.2±2.13 | 14.04±2.80 |
| 193 | Tombarello | Calabria | 70.4±2.71 | 8.54±2.32 |
| 28 | Tonda di Filadelfia | Calabria | 73.7±2.63 | 9.13±1.76 |
| 116 | Tonda di Strongoli | Calabria | 73.2±2.01 | 7.86±0.90 |
| 90 | Tonda Iblea | Sicilia | 69.3± 3.24 | 10.03±1.57 |
| 9 | Tortiglione | Abruzzo | 70±1.5.01 | 11.38±1.20 |
| 70 | Toscanina | Puglia | 76.7± 3.81 | 5.98±3.71 |
| 215 | Tunnulidda | Sicilia | 75.6± 9.71 | 9.72±0.87 |
| 45 | Vallanella | Lazio | 72.4± 4.34 | 8.83±1.61 |
| 123 | Zimbimbo | Puglia | 70.8± 2.13 | 11.15±1.05 |
| 147 | Zinzifarica | Calabria | 67.1± 0.78 | 15.17±1.14 |
