# Supplementary material for: Association Study of the 5′UTR Intron of the FAD2-2 Gene With Oleic and Linoleic Acid Content in Olea europaea L
Source: Front Plant Sci. 2020 Feb 13;11:66. doi: 10.3389/fpls.2020.00066 (PMC7031445; doi:10.3389/fpls.2020.00066)
Supplement: Supplementary file 4 [file Table_2.docx]

**TABLE S2** | Primer list used in amplification reactions of *FAD2-2* fragments.

| FAD2-2-1D F 5’-TGAAGGGCGAGCAGTGTGT-3’  FAD2-2-1co R 5’-ATACAGAACCACCAAACACCAGAC-3’ |
| --- |
| FAD2-2-2co F 5’-GAAATAGAGGTAAAAGATGATAAGTGTGCA-3’  FAD2-2-2co R 5’- TTGAACCAAGAAGAAGCTTCATG -3’ |
| FAD2-2-3co F 5’-GTGGTTGGCCTTTGCTTTTAATC-3’  FAD2-2-3co R 5’-TCAACTCAACAAACACCTTCTGATG-3’ |
| FAD2-2-4Cons F 5’-GGATGTTAGGTTGCAGTCGTCTTAGT-3’  FAD2-2-4Cons R 5’-TGAGAAATATCAACATCTGTAGGCAATC-3’ |
| FAD2-2-5co F 5’-CTTCACATTGCTCATTCGTCATAGAT-3’  FAD2-2-5co R 5’-TCCATCGGATTCCTGATTTGA-3’ |
| FAD2-2-6co F 5’-ATGGAAATACAGTCATCGCCG-3’  FAD2-2-5B R 5’-CAACTCATTTGATCTTCAACAACCA-3’ |
