# Supplementary material for: Association Study of the 5′UTR Intron of the FAD2-2 Gene With Oleic and Linoleic Acid Content in Olea europaea L
Source: Front Plant Sci. 2020 Feb 13;11:66. doi: 10.3389/fpls.2020.00066 (PMC7031445; doi:10.3389/fpls.2020.00066)
Supplement: Supplementary file 5 [file Table_3.docx]

**TABLE S3 |** Primers list for SNPs and IN/DELs analysis in 97 olive cultivars.

| FAD2-2 1N F 5’-GATCTTGTTGTGGGTTGAAGT-3’  FAD2-2 1N R 5’-AATTGCACACTTATCATCTT-3’ |
| --- |
| FAD2-2 2N F 5’-CAATTGTTGAATACAAGATT-3’  FAD2-2 2N R 5’-CTACGCATGTGTTCATATT-3’ |
| FAD2-2 3N F 5’-GGCTAACATGTAGTCCTAGT-3’  FAD2-2 4N R 5’-GACATCAAAATCTATGACGA-3’ |
| FAD2-2 5N F 5’-GATGTCCTACGTCCTATAT-3’  FAD2-2 5N R 5’-AACGGATCGCTGGAAACA-3’ |
