# Supplementary material for: Association Study of the 5′UTR Intron of the FAD2-2 Gene With Oleic and Linoleic Acid Content in Olea europaea L
Source: Front Plant Sci. 2020 Feb 13;11:66. doi: 10.3389/fpls.2020.00066 (PMC7031445; doi:10.3389/fpls.2020.00066)
Supplement: Supplementary file 6 [file Table_4.docx]

**TABLE S4** | List of *cis*-regulatory elements detected in *OeFAD2-2b* intron region.

| **Site Name** | **Sequence** | **Function** |
| --- | --- | --- |
| AAGAA-motif | GAAAGAA | unknown |
| ABRE | CGCACGTGTC | *Cis*-acting element involved in the abscisic acid responsiveness |
| AE-box | AGAAACTT / AGAAACAA | Part of a module for light response |
| ARE | TGGTTT | *Cis*-acting regulatory element essential for the anaerobic induction |
| Box 4 | ATTAAT | Part of a conserved DNA module involved in light responsiveness |
| Box I | TTTCAAA | Light responsive element |
| Box II | AGTCGTGGC | Part of a light responsive element |
| Box-W1 | TTGACC | Fungal elicitor responsive element |
| CAAT-box | CAAT / CAAAT / CCAAT /ggcaat / CAATT | Common *cis*-acting element in promoter and enhancer regions |
| CATT-motif | GCATTC | Part of a light responsive element |
| G-Box | CACGTT / CACGAC / TAACACGTAG | *Cis*-acting regulatory element involved in light responsiveness |
| P-box | CAACAAACCCCTT | Gibberellin-responsive element and light responsive element |
| GA-motif | AAAGATGA | Part of a light responsive element |
| GARE-motif | TCTGTTG | Gibberellin-responsive element |
| GATA-motif | GATAGGA | Part of a light responsive element |
| GCN4_motif | CAAGCCA | *Cis*-regulatory element involved in endosperm expression |
| HSE | AAAAAATTTC | *Cis*-acting element involved in heat stress responsiveness |
| LTR | CCGAAA | *Cis*-acting element involved in low temperature responsiveness* |
| MBS | TAACTG | MYB binding site involved in drought-inducibility |
| MRE | AACCTAA | MYB binding site involved in light-inducibility |
| TATA-box | ATATAT/TATA/TATACA/TTTTA/ | Core promoter element around -30 of transcription start |
|  | TATAAATTAATA/ATATAA/TATAAAT/TAAAAATAA/TATTTAAA/TAATA/TAAAGATT/TTTAAAAA/ TATAAATT/TATAA/ATATAAT |  |
| TCA-element | GAGAAGAATA / CCATCTTTTT | *Cis*-acting element involved in salicylic acid responsiveness |
| Unnamed_4 | CTCC | unknown |
| Circadian | CAANNNNATC | *Cis*-acting regulatory element involved in circadian control |
| AAAC-motif | CAACAAAAACCT | Light responsive element |
| ATGCAAAT motif | ATACAAAT | *Cis*-acting regulatory element associated with |
|  |  | TGAGTCA motif |
| CGTCA-motif | CGTCA | *Cis*-acting regulatory element involved in jasmonic acid responsiveness |
| Skn-1 motif | GTCAT | *Cis*-acting regulatory element related to endosperm expression |
| Sp1 | CC(G/A)CCC | Light responsive element |
| TC-rich repeats | ATTTTCTTCA | *Cis*-acting element involved in defense and stress responsiveness |
| TCT-motif | TCTTAC | Part of a light responsive element |
| TGA-element | AACGAC | Auxin-responsive element |
| TGACG-motif | TGACG | *Cis*-acting regulatory element involved in jasmonic acid responsiveness |
| Unnamed_6 | taTAAATATct | Unknown* |

* elements found in the complement sequence of *OeFAD2-2b*
