# Supplementary material for: Association Study of the 5′UTR Intron of the FAD2-2 Gene With Oleic and Linoleic Acid Content in Olea europaea L
Source: Front Plant Sci. 2020 Feb 13;11:66. doi: 10.3389/fpls.2020.00066 (PMC7031445; doi:10.3389/fpls.2020.00066)
Supplement: Supplementary file 7 [file Table_5.docx]

**TABLE S5** | IMEter results about the intron sequences of *OeFAD2-2* allelic forms and other plant species.

| **Plant species** | **IMEter results** |
| --- | --- |
| *Olea europaea (OeFAD2-2a)* | 18.11 |
| *Olea europaea (OeFAD2-2b)* | 18.24 |
| *Sesamum indicum* | 11.65 |
| *Glycine max* | 11.85 |
| *Arabidopsis thaliana* | 31.75 |
| *Brassica napus* | 11.76 |
| *Perilla frutescens* | 23.25 |
| *Camelina sativa* | 23.85 |
| *Carthamus oxyacanthus* | 42.00 |
| *Carthamus persicus* | 30.19 |
| *Carthamus tinctorius* | 39.24 |
| *Salvia hispanica* | 24.44 |
| *Sinapis alba (FAD2.LIA2)* | 9.89 |
| *Sinapis alba (FAD2.lia2)* | 9.27 |
